# Observations on the Religious Delusions of Insane Persons, and on the Practicability, Safety, and Expediency of Imparting to Them Christian Instruction; with Which Are Combined a Copious Practical Description and Illustration of All the Principal Varieties of Mental Disease, and Its Appropriate Medical and Moral Treatment

**Published:** 1841-07

**Authors:** 


### ART. III.

*Observations on the Religious Delusions of Insane Persons, and on the Practicability, Safety, and Expediency of imparting to them Christian Instruction; with which are combined a copious practical Description and Illustration of all the principal Varieties of Mental Disease, and its appropriate Medical and Moral Treatment.* By NATHANIEL BINGHAM.—*London,* 1841. 8vo, pp. 213.

THE delicate subject of administering religious instruction and consolation to lunatics has been very variously approached by modern physicians; and we fear that the religious excitement prevalent in almost every sect at the present period is not very favorable to its just consideration. Fanatics of every shade and persuasion will rush into extremes, and those who are not fanatics may be provoked into unseemly opposition. Yet the subject is a very serious one, and every medical superintendent of a lunatic asylum must feel the difficulty of determining on the course to be pursued, when error on the one side may cause important religious aid to be withheld, and on the other, a most hurtful excitement to be applied. Some excellent observations on the subject occur in Dr. Burrows's Commentaries on Insanity; but the subject has been apparently avoided by some subsequent writers. The few remarks made by Esquirol on this point are characterized by his usual good sense.

The simple truth seems to be this: as far as religious impressions can be made available to the government of the affections and conduct, they may be usefully encouraged even in a lunatic asylum: if they disturb the mind, they must be avoided, like every other cause of hurtful excitement. This, we suspect would not satisfy Mr. Bingham. He quotes the opinions of Dr. and Mrs. Button, the late surgeon and assistant-matron at Hanwell, to the effect, that lunatics "possess the power of reasoning in every degree, and in the greatest variety;" and that "the difficulty which lies in the way of their instruction arises, not from their want of abilities, but from their dreadful depravity." Mr. Bingham's notes of the opinions of Dr. and Mrs. Button appear to have been made some years ago; and he must surely be incorrect in ascribing such extravagant notions to them. At the time alluded to, nearly the whole staff of Hanwell were reputed to be methodists; but methodists, we imagine, would not accuse all lunatics of dreadful depravity, or assert that they were capable of reasoning in every degree. Sir William Ellis was, we believe, of the methodist persuasion; yet he employs much more reasonable language, far less offensive and unjust to the poor lunatics under his care. The subject of religion was scarcely, if at all, alluded to by him until he made his Fifth Annual Report of Hanwell; wherein he said:

"In former years, from the very incorrect notions entertained of this disease, religious and moral instruction of any kind was never thought of being afforded to the insane. Happily a better knowledge and a better state of feeling now exist. And it is at this time generally admitted that though on some points the mind may be insane, yet on others it may be perfectly rational. And it is no ordinary blessing to many of the sufferers that a just sense of religion often remains, when every other feeling seems obliterated. . . . . Here the patients have the instruction of the Rev. J. Stoddart, the chaplain to the institution, and a more orderly and attentive congregation cannot be assembled together. Some of the committee and other gentlemen have frequently been present, and have expressed their astonishment and delight at witnessing the reverence and decorum of the patients." (Report. Dec. 1835.)

This is moderate and judicious; neither on the one hand containing any insinuation that in unsound mind, the mind is sane on every point, according to the alleged discovery of Dr. Button, nor conveying any libellous sentence upon poor suffering lunatics, as if their depravity was greater than that of the world in which they became mad. In the same reasonable spirit we presume Sir Alexander Morrison to have spoken, on that "fine summer's day" when Mr. Bingham describes himself as walking towards the Middlesex Lunatic Asylum with him; when "a lady of the party" asked Sir Alexander whether he thought the inhabitants of the great house before them were capable of profiting by religious instruction, and "many of them as capable as you or I, was his prompt and emphatic answer." So also Dr. M'Kinnon, of the Aberdeen asylum, when he assured Mr. Bingham that the greater part of his patients attended divine service twice on a Sunday, and that no congregation behaved better, does not seem to have brought this forward as any proof of their dreadful depravity. These words, we confess, haunt us. We should be sorry to think that they were ever seriously used to characterize lunatics by any one still exercising a control over an institution filled with them. We should apprehend that a view of them so limited and illiberal would taint the whole management, and even the whole manner of those entertaining it; and we cannot acquit Mr. Bingham of impropriety as respects recording it, whether Dr. Button be accountable for it or not.

It is rather remarkable that Sir William Ellis did not notice the subject of religious instruction in any of his subsequent reports; remarkable because we believe he was particularly open to religious impressions, and must therefore have abstained from the topic on account of the difficulty of saying what perhaps could alone be said without exaggeration, and at the same time of satisfying those whose ardent expectations in relation to it were not corrected by practical experience.

In the only Report published by Dr. Millingen, the successor of Sir William Ellis, the subject is not alluded to; but in his Aphorisms, which are quoted by Mr. Bingham, he expresses himself rather in a military way concerning it:

"Nothing can be more absurd," says Dr. Millingen, "than the assertion of the great benefit that arises from the patients being obliged to attend divine worship. The apparent tranquillity and attention with which they seem to listen to the chaplain's exhortations are merely mechanical. A lunatic seemed much affected at a sermon, and even shed tears with seeming contrition; the subject of the discourse was the Trinity. When questioned on the homily which thus affected him, he said it was one of the most beautiful sermons he had ever heard, all about the Emperor of Russia and the King of Prussia. Were it not for the moral discipline enforced in these asylums, and the presence of the keepers, their congregations would very frequently exhibit anything but a devotional appearance. During fourteen months' superintendence of Hanwell, out of upwards of one thousand patients, I had only four who were fit to receive religious consolation in sickness and on the bed of death."

As the lunatic congregation at the chapel at Hanwell on a Sunday is probably the largest of the kind in the world, we imagine the further testimony of the superintending officers of that institution down to the present time, although Mr. Bingham has not apparently looked into it, may be considered worthy of attention. In Dr. Conolly's First Report, in October 1839, he says:

"Since the commencement of August, the morning service has been read at eleven o'clock every Sunday, and the evening service read and a sermon preached at half-past three. Each service occupies about one hour. Some of the officers of the establishment invariably attend, and generally about 200 of the patients. A psalm and hymn are usually sung by the congregation, accompanied by the organ; and the responses to the commandments are chanted; by which means the service is so varied as to obviate fatigue or restlessness. The demeanour of the patients on these occasions is for the most part admirable. Few spectacles can be more interesting, or more affecting than that of so many lunatic persons, many of whom are at other times violent, noisy, agitated, and talkative, exercising so remarkable a degree of control over their behaviour for such a length of time. The practice of this control is, unquestionably, the principal advantage which many of the patients are capable of deriving from attendance on these services. Care is taken that they appear decently dressed; several of those who can read are supplied with prayer-books; and they evidently look forward to Sunday with pleasure; and are mortified when any accident interferes with their attendance in chapel. Yet a very small number of them seem to have any distinct religious impressions. Many are prone to the terrors of an alarmed conscience, and believe that evil spirits are immediately busy around them. A few present examples of religious conceit; several consider themselves to be divine persons. One asserts that he is the Almighty; and refuses to go to chapel, although he rings the chapel bell very diligently. In very few of the patients does religion appear to be a source of hope and tranquillity. The cautions which these circumstances render necessary in the attempt to administer religious instruction to them are too obvious to require to be dwelt upon."

The subject is again touched upon in the Second Report of the same physician, after the additional experience of another year:

"About 300 patients have attended at chapel twice every Sunday during the year. No familiarity with this spectacle can weaken its impressive effect. Occasional oddities of conduct and slight irregularities are displayed; but the general demeanour of the congregation is most remarkable.

"It happens almost every week that patients who are recovering from attacks of excitement, are petitioners for the indulgence of going to chapel. Whenever there is reason to think they may be trusted, their desire is complied with; and, generally speaking, they entirely control their conduct during the service. Exceptions of course sometimes occur; so many irritable beings being collected together.

"Among the old and incurable patients many are constant attendants at chapel; and, with respect to several of them, no manifestation of insanity is made which would lead a person unacquainted with them to conjecture that they were lunatics. Among the men, there are many examples of this kind; and the gravity and respectability of their appearance would cause an observer to be extremely struck with the contrast presented by their incoherent conversation when spoken to. Some of the men and women also, who would be deeply mortified by being excluded from chapel, practise a few eccentricities when there, but generally of so harmless a kind, that to exclude them would seem to border on cruelty. So accustomed are the patients to preserve their composure during the hour of service, that if, as sometimes happens, an epileptic patient utters a loud scream, falls into a fit, and requires to be taken out by the keepers or nurses, very few of the patients quit their seats; and those in the immediate neighbourhood of the person affected usually render what assistance they can, and then quietly resume their places.

"Although so many male and female patients attend the chapel regularly, the physician has only found it practicable to recommend a very small number to the private attention of the chaplain. Whenever a desire is expressed to see him it is complied with; and to those who are seriously ill, it is often suggested that they should have some conversation with him. This is sometimes declined, but more commonly accepted. To make similar propositions to the numerous patients who think that nothing whatever is the matter with them, would probably give rise to morbid trains of ideas, which it is better not to excite. There are also some patients in the asylum whose thoughts perpetually dwell on religious topics; but with so much wildness and enthusiasm as to render it prudent not to encourage but rather to avert such ideas from their minds. Some of the patients admitted with a propensity to suicide, have appeared to be comforted by conversation with the clergyman. A great number of the patients are gratified by being allowed to have a Bible and Prayer-book."

We have quoted the preceding passages with the more satisfaction, because whilst they betoken no indifference to the subject of religion, they evince no signs of temporising in a matter on which to speak without cant is often to incur a great weight of theological hatred. We not long ago visited an asylum in which the chaplain, dissatisfied with the simple restriction of his visits to the patients who wished to see him, straightway proclaimed one of the officers to be a Unitarian, another an Infidel, a third a Socialist, and all the rest the church's enemies. In that asylum we listened, with dismay, to emphatic assurances to the melancholy and the mad that the devil was ever seeking to devour them. We heard them instructed in the reason wherefore in the bottomless pit they would be bound with chains instead of cords, (a sort of restraint-simile); namely, because the chains were the stronger; and for equally sound reasons they were given to understand that their sins were written not in common ink, but in marking ink, which would not wash out. They were invited, God help them! to the Lord's supper, not in our Lord's affectionate and affecting words, "Do this in remembrance of me;" but in the fierce language in which St. Paul perhaps not undeservedly addressed the Romans, when he told the sacrificers to Mars and Venus, who affected also to be Christians, that they could not partake of the Lord's cup and of the cup of devils! We were happy, at the time, to think that the poor denounced mad people were insensible to these insults; which, if they had possessed the exquisite reasoning power spoken of as belonging to them, must deeply have stung them, notwithstanding their "dreadful depravity." We dare say that precedents for all this strong language may be cited from the older divines; but our remarks apply to it as addressed to lunatics. It may be necessary to awaken hardened sinners by hard words; but the error seems to be that of confounding insane persons with hardened sinners.

Every reasonable person will see that the subject should be calmly entered upon; and we hope that in proportion to every medical man's anxiety for every lunatic to enjoy spiritual as well as all other advantages, will be his exertion to prevent such attempts to intimidate lunatics on religious matters, as would really be madder than madness itself. Mr. Bingham has, we think, entered on the subject with strong prepossessions, in consequence of which he sets aside, as he himself states, the testimony "of a medical friend, who has seen as many insane patients as any gentleman in the kingdom;" and who told him that he had not "witnessed among them the good effect of religious instruction;" a gentleman, too, who does not question its propriety, but who doubts its being practicable to make a salutary impression among them. In the same way Mr. Bingham avows that he rejects the opinion of another witness from a quarter where he knows "the experiment has been long tried on the usual plan." This at least shows considerable adhesiveness in Mr. Bingham to his own opinion; which he at the same time affirms to be based on extensive observation. He thinks, without any perceptible reason for so thinking, as it appears to us, that the religious services in asylums are too generally merely formal; and even that patients are discouraged from attending. But we agree with him that, although the effect of a sermon may be inconsiderable, that of occasional short conversations may be beneficial; and he is perfectly correct in stating that many of the patients in asylums have been left all their lives in the grossest ignorance of religious truths. We can also readily conceive his eagerness to profit by their situation, if possible, "before they are sufficiently well to be discharged." But they must be affectionately addressed; they must love and respect their pastor; he must assume no airs of secular superiority; he must refrain from the scolding tone of one preaching to hardened convicts; and he must have sense and discretion enough to know when to desist, and when to hold his peace. If he has not this sense and discretion, and much real benevolence also, his labours will be vain, and his interference mischievous. The great principle in the government of lunatics is to refrain from irritating them; and, on every occasion, if not quite impossible, to proceed by methods of persuasion instead of force. If a few words in season are beneficial, a very few words out of season are equally pernicious. When these precautions are not despised, the chaplain may become a highly useful officer; and if he is a man of sense he will not consider it any degradation to be an auxiliary to the physician. Then it may be really ascertained in what proportion and description of cases the insane patient was capable of listening with patience and edification to religious conversation; and how much advantage the means of cure may receive from this exalted means. This will never be ascertained by those who consider the inmates of a lunatic asylum, or even those of them who delight in attending the religious services, as quite as capable of receiving the attention of a clergyman as any other persons of their class of life. The very maintenance of such ground would prove that the clergyman wanted the knowledge requisite for the office he had undertaken. But if, in addition to this want of knowledge, he should unhappily imbibe the notion of their "dreadful depravity," no more mischievous person could possibly be admitted into the wards. Indeed everything depends on the choice of a chaplain; and the attention paid to his qualifications cannot be too careful. When the choice is injudicious, the physician has an enemy within his gates, to whom to yield is to abandon his duty, and with whom to contend is to expose himself to imputations of disrespect for the clergy, the church, and even religion itself.

Mr. Bingham's sensible remarks on the Moral Treatment convince us that he would himself observe the utmost care in applying religious means to lunatics; and he seems perfectly aware that there are some to whom such means could not be applied, and some to whom they might do mischief. He appears to us, to say the truth, when applying himself to the practical consideration of the subject, to have corrected his own views. At all events his remarks on the Mode of conducting the Religious Instruction of the Insane are characterized by judgment as well as earnestness. He observes, that the whole household of a lunatic asylum should unite in the attempts to make religious instruction or religious services acceptable to the patients; and that the spirit of the place should not be sectarian, but that which is preserved in the bond of peace. He lays down very good precepts touching the duties of the chaplain; whose office he regards as one enabling him materially to aid the superintendent, and to give his undivided attention to doing good. The suggestion of classing the more religious patients together, under a "pious keeper," is one which it would probably be found difficult to carry into effect; and the various means, admirable in themselves, which Mr. Bingham suggests as promotive of religious feelings in the insane, require the utmost good sense on the part of those endeavouring to apply them. Mr. Bingham does not overlook the frequent occurrence of religious despondency among the insane, and the cautious conduct to be observed towards those thus affected. He at the same time points out the common error of considering all cases in which the insanity is manifested by excessive devotion and enthusiastic religious talk, as cases originating in religious enthusiasm; showing that the devotional character is often only a part of the insanity, and disappears entirely on the patient's recovery from the disease; the malady itself arising from some disorder of the stomach or bowels. His opinion is, however, that the temporary religious feeling may be improved into a salutary habit, if the patient has the advantage of a minister or a religious friend. Mr. Bingham is, at the same time, quite explicit on the subject of other cases, in which excessive religious zeal has passed into insanity, and in which he acknowledges the necessity of withdrawing books, preachers, and injudicious friends. He does not, even in these cases, advise a total discouragement of religious ideas; by which he conceives the patient might be led to despair. In cases of melancholia he quotes Sir William Ellis and Dr. Conolly as having witnessed the good effects of music; and although such effects are probably not very durable, the frequent appliance of this and other means of promoting cheerfulness may be more permanently effectual. Reasoning, Mr. Bingham well observes, is of very little service in the case of delusions, whether religious or of any other kind; and the best thing is to lead the mind away from the delusive subject as much as possible. This is far preferable even to appearing to coincide in such delusions. They should seldom be alluded to; be contradicted calmly, if at all; and never ridiculed. Thus managed, they will often die away of themselves. Here, as in all parts of the treatment of insanity, there is wisdom in that avoiding of irritation of which we have already spoken. "Patients," says Mr. Bingham, "are sometimes *suddenly* set free from their illusions, but they are never talked out of them." In some instances of melancholy, he is of opinion that even the positive *excitement* of religion may be usefully tried; and the doctrines of Christianity preached with impassioned eloquence with good effect.

Upon the whole, there is so much that is deserving of cordial approval in Mr. Bingham's little work, that we feel justified in recommending it to all who have the care of the insane. The whole subject is attended with peculiar difficulty, and should be met in a candid, earnest, and philanthropic spirit. Above all things, let the attempt to extend religious instruction to lunatics be animated by a due consideration of their state and character; their state, the most pitiable that is incidental to God's reasonable creatures; and their character, not as spoken of by some of Mr. Bingham's witnesses, dreadfully depraved; but such as described by one who knew them well, and served them faithfully, and described them truly:—and whose words are quoted by Mr. Bingham. It is thus that Pinel speaks of them:

"I cannot here avoid giving my most decided testimony to the moral qualities of maniacs. I have nowhere met, except in romances, with fonder husbands, more affectionate parents, more impassioned lovers, more pure and exalted patriots, than in the lunatic asylum, during their intervals of calmness and reason. A man of sensibility may go there every day of his life, and witness scenes of indescribable tenderness associated to a most estimable virtue.

"Let us then approach them with kindlier feelings than have been usually cherished. Let us treat them as friends and as children. Let everything possible be done to alleviate their sufferings, to console and make them happy. And if we cannot win them over to piety, let them not have to say that our harsh conduct has confirmed their dislike to our principles."

---

